# Laparoscopic Cholecystectomy in a Patient With Situs Inversus Totalis Presenting With Cholelithiasis: A Case Report

**DOI:** 10.3389/fsurg.2022.874494

**Published:** 2022-04-14

**Authors:** Tao He, Jieyu Zou, Haiyu Song, Bin Yi, Ke Sun, Juan Yang, Tingting Lei, Lin Xu, Guangkuo Li

**Affiliations:** ^1^Department of Hepatobiliary Surgery, Chengdu Second People's Hospital, Chengdu, China; ^2^The Second Affiliated Hospital of Chongqing Medical University, Chongqing, China

**Keywords:** situs inversus, cholelithiasis, laparoscope, cholecystectomy, case report

## Abstract

Laparoscopic cholecystectomy is the standard treatment for cholelithiasis. A very rare condition named situs inversus should not be considered as a contraindication for laparoscopic cholecystectomy. Here, we reported a case of successful laparoscopic cholecystectomy in a patient with situs inversus totalis. We also described the technical advantages of this treatment and reviewed the literature.

## Introduction

Situs inversus is a congenital developmental anomaly of the positioning of the internal viscera which is divided into partial and total visceral inversion (1). The incidence of situs inversus totalis (SIT) ranges from 1:10,000 to 1:20,000 (2). The patient with cholelithiasis has presented differently, wherein they have left upper abdominal pain or discomfort leading to delayed diagnosis. As the gold standard technique for removal of pathological gallbladder, performing laparoscopic cholecystectomy with situs inversus will induce great challenges right from the diagnosis, investigating the patient to the most important aspect, i.e., performing the procedure itself. Here, we shared a case of a patient with cholelithiasis, along with recurrent upper left pain as the dominant presentation, who resoundingly underwent laparoscopic cholecystectomy by changing the position of the operation.

## Case Report

**Figure 1:**
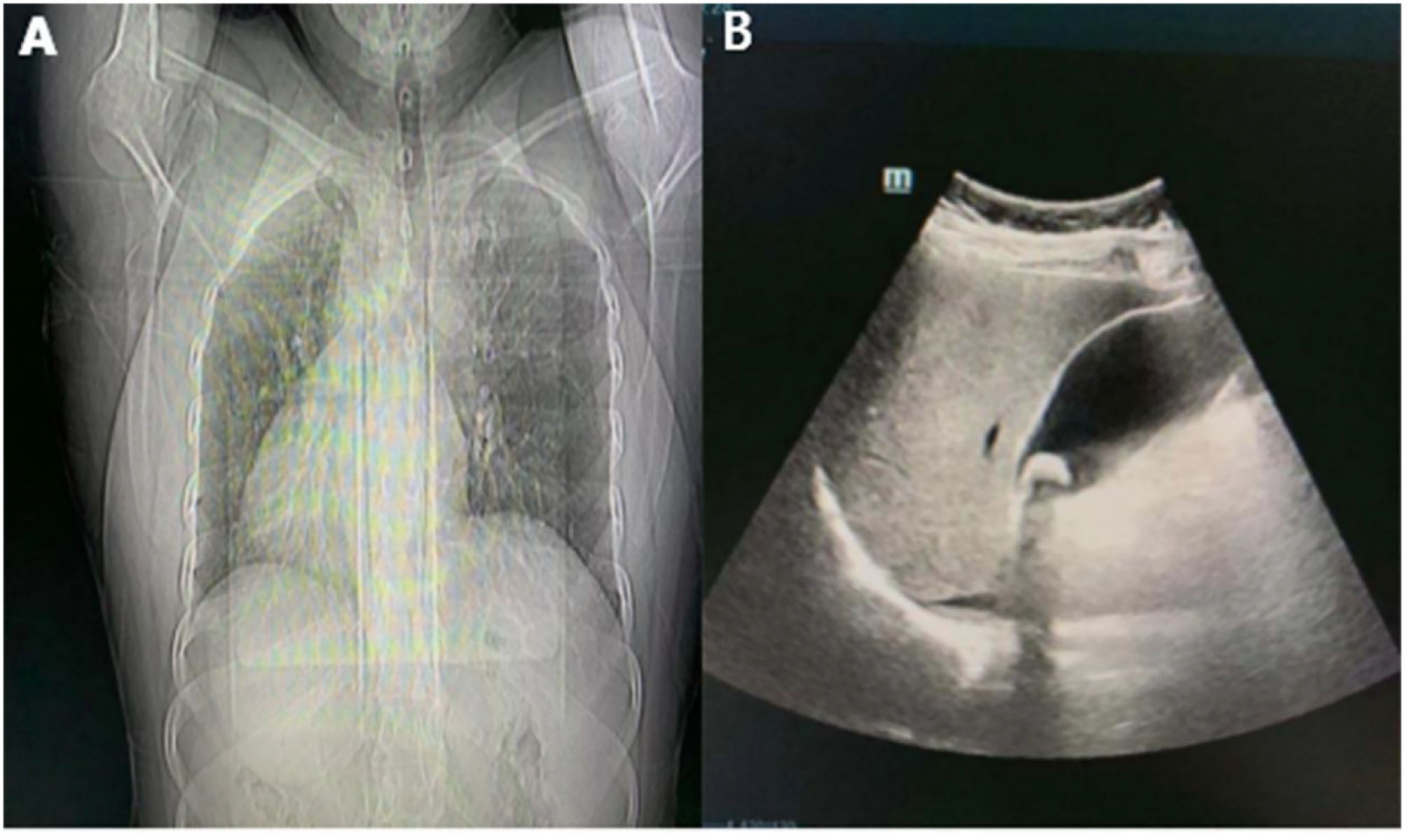
**(A)** Chest X-ray displays dextrocardia; **(B)** ultrasound indicated the gallbladder was located in the left upper abdomen with cholecystolithiasis.

**Figure 2:**
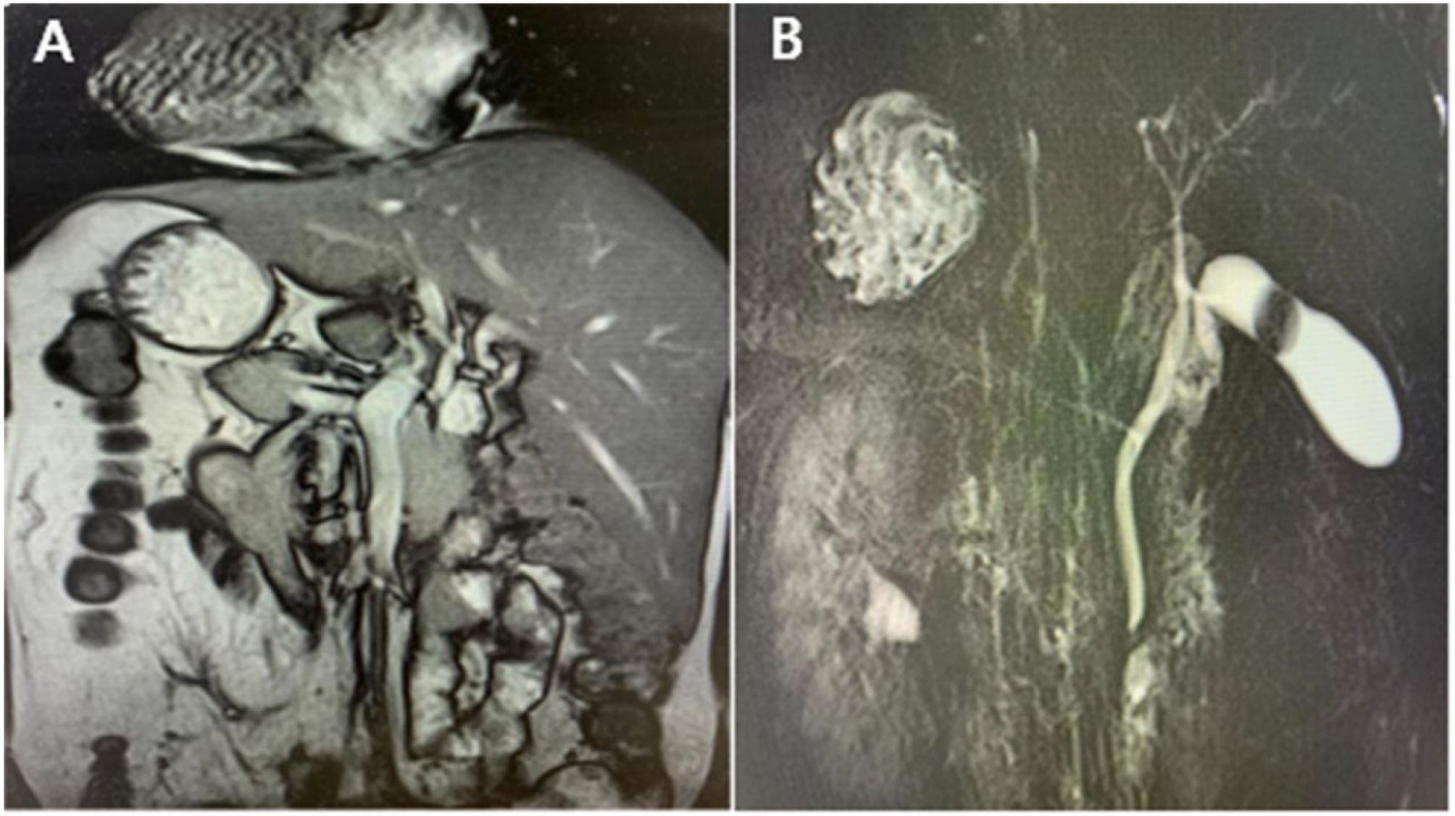
**(A)** Situs inversus totalis **(B)** ectopic gallbladder and cholelithiasis.

A Tibetan patient who is a 53-year-old woman was admitted to the hospital due to intermittent left upper abdominal pain for more than 1 year and eventually worsened for 7 days, beginning on December 9, 2021 onwards. They had no fever, jaundice, or vomiting. Regrettably, they just took analgesics instead of seeking medical attention. Their past medical history revealed open appendectomy and SIT. Besides that, they do not have any family medical and psychosocial history. Abdominal examination showed a soft abdomen and left epigastric tenderness, without rebound tenderness or muscle tension. The results of their laboratory examination, including routine analysis of blood, liver renal function, or coagulation were normal. There were no signs of elevation of amylase in blood and urine. Dextrocardia was displayed in chest X-rays. Abdominal ultrasound suggested cholelithiasis and an enlarged gallbladder in the left upper quadrant (Figure 1). The patient underwent abdominal magnetic resonance cholangiopancreatography (MRCP), which indicated cholelithiasis, with no evidence of dilated bile duct (Figure 2). Considering the presence of dextrocardia and situs inversus, the patient received cardiac and respiratory function tests before surgery. Color Doppler echocardiography indicated dextrocardia and no significant structural or functional abnormalities. Lung function tests showed no obvious lesions. After discudding in the department, doctors considered cholelithiasis and ruled out the diagnosis of acute pancreatitis based on the patient's clinical presentation and related ancillary tests (without pancreatic edema and amylase activation). It was concluded that the patient had clear indications for surgery and no contraindications. Laparoscopic cholecystectomy was performed on the third day after admission. Histopathology of sections from the gallbladder revealed features suggestive of chronic cholecystitis. The patient was discharged on the third postoperative day and no special discomfort was observed until January 10, 2022.

**Figure 3:**
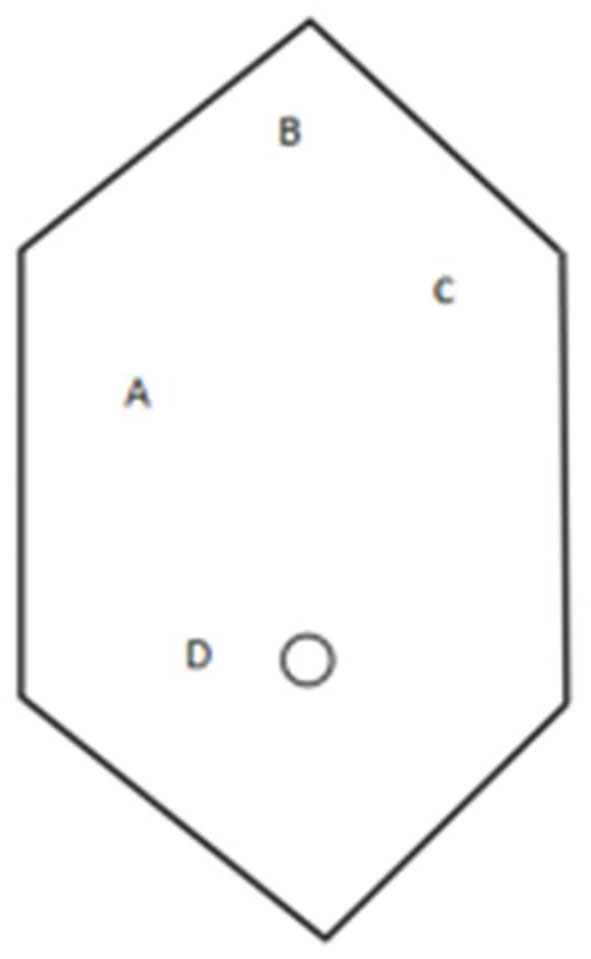
The layout of this case (A: main operation, B and C: deputy hole, and D: camera port).

In our case, the patient was in the supine position, with their head up and feet down at an angle of 30° and leaning on 20° left. We set up carbon dioxide (CO2) pneumoperitoneum pressure at 14 mmHg, and the camera port was inserted on the right side, ~2 cm away from the umbilicus. The location of the main operation hole, used by 10 mm trocar, was about 5 cm at the lower edge of the intersection of the right midline of the clavicle and costal margin. Two additional points were, respectively, selected at the right of the lower xiphoid process and below the left costal margin about 2 cm through the assistant (Figure 3). The surgeon with strong left-handed operation ability used an ultrasonic scalpel through a hole to separate the adhesions, which were caused by the gallbladder, duodenum, and liver, and fully exposed the common bile duct, cystic duct, and cystic artery. Fortunately, there was no variation in the bile duct system. There were multiple pigmented calculi in the gallbladder without pus. Meanwhile, mucosal atrophy can be discovered in specimens and sent for pathological examination after surgery. The operation took 60 min.

## Discussion

Situs inversus totalis is an extremely rare congenital disorder which means the internal organs are completely opposite to those of the normal body. However, the physiological function of the organ is consistent with that of ordinary people. Elbeshry et al. (3) studied SIT, autosomal genetic disease, which may be due to abnormal parental genes that may have aberrant translocation during embryo development. Although the cause of the disease is unknown, a slightly higher incidence has been reported in women (4). Because of the change of position, the presentation of acute cholecystitis resembles that of acute pancreatitis, which significantly increases the risk of missed diagnosis. Ultrasound may be one of the easiest and quickest ways to make a diagnosis.

The laparoscopic technique of SIT has been widely reported in appendicitis and sleeve gastrectomy (5, 6). Meanwhile, surgeons revealed a variety of layouts by changing the position of torcars (7). Campos and Sipes (8) firstly reported a 39-year-old woman with SIT who successfully underwent laparoscopic cholecystectomy in 1991. They took cholangiography demonstrated the inverted position of the bile duct and duodenum and ruled out the presence of extrahepatic bile duct tree stones. Meanwhile, the surgeon and the assistant placed a four-port technique using a “mirror image” port placement technique for conventional laparoscopic cholecystectomy. Through this approach, surgeons have comprehended how laparoscopic cholecystectomy is performed in SIT. However, this technology is not friendly to most right-handed surgeons. Meng et al. (9) performed a three-port way using two 10 mm trocars in the lower edge of the umbilical cord, while in the upper edge, 10 cm and 5 mm trocar is placed to the left, below the xiphoid process to form the auxiliary operating hole. Although this method reduces one operation hole, it has high requirements for the surgeon's left-hand operation ability. Aside from that, Hu et al. (10) received a patient with choledocholithiasis and gallbladder stones and completed a four-port laparoscopic cholecystectomy like Campos and Sipes (8) after endoscopic retrograde cholangio pancreatography (ERCP). However, the surgeon and the mirror-gripping assistant were on the left side of the patient, and the first assistant was on the right side.

In our patient, we also choose a four-port method and supported by some advantages. The surgical team also changed positions, with the surgeon and second assistant standing on the right and the first assistant on the left. In this case, the anatomy was unclear due to adhesion around the gallbladder caused by a previous open appendectomy. Thus, the chirurgeon employed the ultrasonic scalpel to separate the adhesion and adipose tissue, and the assistant fully revealed the gallbladder triangle by holding the bottom of the gallbladder and pulling it left and up. In this way, it is easier for surgeons to adopt the right hand, make the anatomy clearer, and shorten the operation time. Indeed, this method requires the surgeon to have strong hands-on skills by using the energy instrument with the adverse hand, which may be the main disadvantage in this way.

## Conclusion

The ectopic gallbladder is not a contraindication for laparoscopic cholecystectomy. The position of trocars in SIT should be adjusted appropriately to achieve a clear gallbladder triangle through preoperative judgment. We believe that this technique will allow right-handed surgeons to perform it proficiently in this particular situation. Besides, patients may benefit from this minimally invasive technique.

## Ethics Statement

Written informed consent was obtained from the individual(s) for the publication of any potentially identifiable images or data included in this article.

## Author Contributions

TH and JZ: article writing. HS and BY: performed image acquisition. TH, KS, TL, and JY: data collection. TH, LX, and GL: revised and improved the article. All authors contributed to the article and approved the submitted version.

## Conflict of Interest

The authors declare that the research was conducted in the absence of any commercial or financial relationships that could be construed as a potential conflict of interest.

## Publisher's Note

All claims expressed in this article are solely those of the authors and do not necessarily represent those of their affiliated organizations, or those of the publisher, the editors and the reviewers. Any product that may be evaluated in this article, or claim that may be made by its manufacturer, is not guaranteed or endorsed by the publisher.

## References

[1] Akbulut S. Comment on “a systematic review of laparoscopic cholecystectomy in situs inversus”. J Invest Surg. (2021) 34:637. 10.1080/08941939.2019.1673854 31790321

[2] Shah AY Patel BC Panchal BA. Laparoscopic cholecystectomy in-patient with situs inversus. J Minim Access Surg. (2006) 2:27–28. 10.4103/0972-9941.25674 21170224 PMC2997218

[3] Elbeshry TM Ghnnam WM. Retrograde (fundus first) laparoscopic cholecystectomy in situs inversus totalis. Sultan Qaboos Univ Med J. (2012) 12:113–5. 10.12816/0003097 22375268 PMC3286706

[4] Pitiakoudis M Tsaroucha AK Katotomichelakis M Polychronidis A Simopoulos C. Laparoscopic cholecystectomy in a patient with situs inversus using ultrasonically activated coagulating scissors. Report of a case and review of the literature. Acta Chir Belg. (2005) 105:114–7. 10.1080/00015458.2005.11679682 15790219

[5] Akbulut S Ulku A Senol A Tas M Yagmur Y. Left-sided appendicitis: review of 95 published cases and a case report. World J Gastroenterol. (2010) 16:5598–602. 10.3748/wjg.v16.i44.5598 21105193 PMC2992678

[6] Yazar FM Emre A Akbulut S Urfalioglu A Cengiz E Sertkaya M. Laparoscopic sleeve gastrectomy in situs inversus totalis: a case report and comprehensive literature review. Indian J Surg. (2016) 78:130–5. 10.1007/s12262-015-1437-y 27303123 PMC4875906

[7] Akbulut S Caliskan A Ekin A Yagmur Y. Left-sided acute appendicitis with situs inversus totalis: review of 63 published cases and report of two cases. J Gastrointest Surg. (2010) 14:1422–8. 10.1007/s11605-010-1210-2 20567931

[8] Campos L Sipes E. Laparoscopic cholecystectomy in a 39-year-old female with situs inversus. J Laparoendosc Surg. (1991) 1:123–25. 10.1089/lps.1991.1.123 1834260

[9] Meng Y Guo H Peng J Zhang X Yang X. Modified laparoscopic cholecystectomy for cholelithiasiswith situs inversus totalis: a case report. Asian J Surg. (2022) 1. 10.1016/j.asjsur.2021.12.005 35000850

[10] Hu L Chai Y Yang X Wu Z Sun H Wang Z. Duodenoscope combined with laparoscopy in treatment of biliary stones for a patient with situs inversus totalis: a case report. Medicine. (2019) 98:e14272. 10.1097/MD.0000000000014272 30762727 PMC6408073

