# Supplementary material for: Laparoscopic Cholecystectomy in a Patient With Situs Inversus Totalis Presenting With Cholelithiasis: A Case Report
Source: Front Surg. 2022 Apr 14;9:874494. doi: 10.3389/fsurg.2022.874494 (PMC9046872; doi:10.3389/fsurg.2022.874494)
Supplement: Supplementary file 1 [file Table_1.DOCX]

Supplemental table results of general laboratory tests on admission

| Item | results |
| --- | --- |
| WBC | 5.4x109/L |
| NEUT | 2.72x109/L |
| NEUT% | 50.30% |
| Hb | 147g/L |
| HCT | 43.70% |
| PLT | 238x109/L |
| PT | 10.4s |
| INR | 0.95 |
| FDP | 1.1ug/ml |
| ALT | 17U/L |
| AST | 22U/L |
| ALb | 43.6g/L |
| TBil | 5.8umol/L |
| DBil | 3.9umol/L |
| AFP | 1.4ng/ml |
| CEA | 1.27ng/ml |
| PCT | <0.02 |

NEUT:[neutrophile](javascript:;) [granulocyte](javascript:;),TBil:[total](javascript:;) [bilirubin](javascript:;),IBil:[direct](javascript:;) [bilirubin](javascript:;)

PT:[prothrombin](javascript:;) [time](javascript:;),FDP:fibrinogen degradation product
